# Renal Complication and Glycemic Control in Korean Veterans with Type 2 Diabetes: A 10-Year Retrospective Cohort Study

**DOI:** 10.1155/2020/9806790

**Published:** 2020-06-22

**Authors:** Ye An Kim, Young Lee, Je Hyun Seo

**Affiliations:** ^1^Division of Endocrinology, Department of Internal Medicine, Veterans Health Service Medical Center, Seoul 05368, Republic of Korea; ^2^Veterans Medical Research Institute, Veterans Health Service Medical Center, Seoul 05368, Republic of Korea

## Abstract

**Objective:**

Tight glycemic control reduces the risk of diabetes complications, but it may increase the risk of hypoglycemia or mortality in elderly patients. This study is aimed at evaluating the incidence and progression of renal complications and its association with glycemic control in elderly patients with type 2 diabetes.

**Methods:**

This retrospective cohort study examined the data of 3099 patients with type 2 diabetes who were followed for at least 10 years at the Korean Veterans Hospital and for whom glycated hemoglobin (HbA_1c_) was measured in 2008 and 2017. Participants were divided into six groups according to their baseline or dynamic HbA_1c_ levels. Extended Cox models were used to calculate adjusted hazard ratios for the development of chronic kidney disease (CKD) and end-stage renal disease (ESRD) associated with specific HbA_1c_ ranges.

**Results:**

During the 10-year follow-up period, 30% of patients developed new CKD, 50% showed progression, and ESRD developed in 1.7%. The risk of CKD was associated with baseline HbA_1c_ from the first year of the study and dynamic HbA_1c_ throughout the study period. The adjusted hazard ratios for CKD were 1.98 and 2.32 for baseline and dynamic HbA_1c_, respectively, at the level of ≥69 mmol/mol. There was no increased risk for any complications in baseline and dynamic HbA_1c_ below 58 mmol/mol.

**Conclusions:**

A higher HbA_1c_ ≥ 58 mmol/mol was associated with an increased risk of diabetes complications. A less stringent glycemic target of HbA_1c_ could be used as the threshold of renal complications.

## 1. Introduction

Type 2 diabetes is a chronic metabolic disorder characterized by hyperglycemia due to defects in insulin secretion, action, or both [1, 2]. In 2015, approximately 415 million people worldwide had diabetes, including 230 million Asians [3], and this is projected to increase to 642 million people worldwide and 355 million Asians by 2040. The rapidly growing incidence of diabetes is a serious problem [3]. Despite the high prevalence of diabetes in the general and elderly population, the appropriate glycemic goal remains controversial because large-scale longitudinal studies for diabetic complications, such as nephropathy, are lacking.

The widely accepted glycated hemoglobin (HbA_1c_) goal of <53 mmol/mol (7.0%) is based on the results of the Diabetes Control and Complication Trial and UK Prospective Diabetes Study (UKPDS), which showed that HbA_1c_levels < 53 mmol/mol (7.0%) reduced the risk of microvascular complications [4–6]. Based on these findings, many organizations, including the Korea Diabetes Association, recommend maintaining a target HbA_1c_ < 48 mmol/mol (6.5%) for the general population with type 2 diabetes patients, and 53 mmol/mol (7.0%) for type 1 diabetes patients or elderly type 2 diabetes patients. However, although it was still effective in preventing the development and progression of nephropathy and albuminuria, intensive glycemic control failed to show a protective effect in reducing cardiovascular complications in elderly patients with longstanding diabetes or cardiovascular risks [7–9]. Furthermore, the Action to Control Cardiovascular Risk in Diabetes (ACCORD) trial revealed a higher mortality rate in the intensive glucose-lowering treatment group [9]. Subsequent observational studies have also revealed conflicting results regarding optimal glycemic targets [10–12].

Chronic kidney disease (CKD) is one of the major complications of diabetes. It occurs in approximately one-third of patients with diabetes [13]. In the Veterans Affairs Diabetes Trial (VADT) [14], intensive glycemic control did not significantly affect the progression of renal disease in the entire cohort except in the high-risk groups. However, a recent meta-analysis showed that intensive glucose control over 5 years reduced both kidney and eye events [15].

CKD and diabetes reinforce the risk of cardiovascular disease [16]; thus, given the increasing prevalence of diabetes and longstanding diabetes, the burden of CKD might increase in the future. Data for determining the appropriate HbA_1c_ level to prevent CKD in specific elderly patients with type 2 diabetes are limited. Thus, this study is aimed at investigating the association between target HbA_1c_ and the risk of CKD development and its progression in elderly patients with longstanding diabetes. Towards this goal, we conducted a large-scale analysis of a Korean Veterans Diabetes cohort.

## 2. Materials and Methods

### 2.1. Subjects

This was a retrospective cohort study of patients with type 2 diabetes in the outpatient clinic of the Veterans Health Service Medical Center (Seoul, Korea). Since electronic medical records (EMR) were first introduced in this hospital in 2008, we screened patients who had been followed for at least 10 years and whose HbA_1c_ values were measured both in the first year (2008) and in the last year (2017) of the study. The inclusion criteria were as follows: (1) outpatients aged 18 years or older who visited the endocrinology department with type 2 diabetes (defined using the ICD-10 codes E11.0–11.9) in 2008 and (2) first HbA_1c_ ≥ 48 mmol/mol (6.5%) or at least taking antidiabetic medication. Further, most study subjects were regular patients at the outpatient clinic. At the beginning of the study, patients with ESRD were excluded, because they no longer showed a progression of renal complications or the development of ESRD, which are the primary endpoints of the current study. Of the 3648 patients identified, we excluded 549 patients with evidence of end-stage renal disease (ESRD) before the baseline period (*n* = 2), those who had missing data (serum creatinine and urine protein within 2 years of the initial and last year of the study period; *n* = 316), and those who had malignancies affecting diabetes progression (stomach, pancreas, and kidney; *n* = 231). Finally, 3099 patients were included. Available data of these patients were extracted from the Veterans Hospital Medical Information System (ezCaretech, Korea) using a clinical data warehouse. This study was approved by the Institutional Research Committee (IRB No. 2017-11-002), and the need for informed consent was waived owing to the retrospective study design.

#### 2.1.1. HbA_1c_ Categories and Variables

Because the existing target HbA_1c_ levels have been suggested in 0.5% increments, the patients were divided into six groups according to their baseline or median dynamic HbA_1c_ levels as follows: <48 mmol/mol (6.5%), 48–53 mmol/mol (6.5–7.0%), 53–58 mmol/mol (7.0–7.5%), 58–64 mmol/mol (7.5–8.0%), 64–69 mmol/mol (8.0–8.5%), and ≥69 mmol/mol (8.5%). Dynamic HbA_1c_ was defined as the HbA_1c_ values, measured at approximately two-year intervals during the follow-up period or until the events occurred. HbA_1c_ were measured by high-performance liquid chromatography (HLC-723G7; Tosoh, Tokyo, Japan) certified by the National Glycohemoglobin Standardization Program (NGSP). Baseline covariates were obtained within the first 2 years of study and included demographics, such as age and sex. Body mass index (BMI) was calculated as weight (kg) divided by height^2^ (kg/m^2^). Blood pressure was measured using an automatic manometer. Blood samples were obtained after at least 8 hours of fasting. High-density lipoprotein (HDL) cholesterol, low-density lipoprotein (LDL) cholesterol, triglycerides, uric acid, and creatinine were measured using an enzymatic colorimetric method (Toshiba Medical System Co. Ltd., Tokyo, Japan). As an indicator of renal complication, serum creatinine and urine protein levels were measured 1.9 times per year and 1.47 times per year on average, respectively, over the entire study period. The estimated glomerular filtration rate (eGFR) was calculated using the abbreviated Modification of Diet in Renal Disease equation as follows: 175 × serum creatinine^−1.154^ × age^−0.203^ (×0.742 if female) [17]. Baseline comorbidities and other diabetes complications were extracted from the electronic medical records throughout the study period defined by the following ICD-10 codes: hypertension (I10.9–11.0), hyperlipidemia (E78.0–78.5), ischemic heart disease (I20.0–24.9), heart failure (I50.0–51.9), cerebrovascular disease (I60.0–69.0), diabetic retinopathy associated with type 2 diabetes mellitus (E11.30–11.38), type 2 diabetes with neuropathy (E11.28–11.42), and diabetic foot (E11.7 and E14.8). Prescription data for statin, antihypertensive agents, and glucose-lowering agents were investigated, and the baseline medication was included if the drug was started within 3 years from the start of the study.

#### 2.1.2. Assessment of Renal Complications

Renal complication was assessed using eGFR and albuminuria categories according to the Kidney Disease: Improving Global Outcomes (KDIGO) guidelines [18]. CKD development and progression were also defined according to the KDIGO guidelines. CKD stage 1–2 with normal to mildly increased albuminuria was considered as a normal renal function (CKD-naïve during the initial study period). New CKD development was defined as having an eGFR < 60 mL/min/1.73 m^2^ or albuminuria > 3 mg/mmol over 3 months in CKD-naïve patients. CKD progression was defined as a decline in the eGFR category without reversion. Due to the variable amount of albuminuria, a decline in albuminuria category was considered as increased, but not interpreted as CKD progression. As defined, CKD progression was possible only in preexisting CKD patients (CKD stage 3A, 3B, 4, and/or albuminuria at moderately or severely increased levels). The time of CKD progression was calculated as the time at which the stage was deteriorated owing to a decreased eGFR in patients with CKD stage 3A, 3B, or 4 at the beginning of the study. ESRD development was defined as an eGFR < 15 mL/min/1.73 m^2^. Based on initial values, subjects were divided into two groups as the CKD-naïve group and the preexisting CKD group, and changes in renal function were estimated. The proportion of patients who developed CKD in the CKD-naïve group and CKD progression in the preexisting CKD group was analyzed with the final eGFR and albuminuria category in the last observation at 10 years. In the survival analysis, the renal outcome was defined as a composite of CKD development in the CKD-naïve group and CKD progression in the preexisting CKD group. Because the group with normal GFR with micro-/macroalbuminuria was neither CKD naïve nor CKD, by definition, it was not included in the CKD analysis. In contrast, it was included in the ESRD analysis, which was performed in all subjects.

### 2.2. Data Analyses

The characteristics of the enrolled patients were analyzed based on the six baseline HbA_1c_ categories. Categorical data were analyzed using Fisher's exact or Chi-square tests. One-way analysis of variance was used for continuous variables, and Tukey's test was used as a post hoc test. Survival analysis was initiated from the day of first HbA_1c_ measurement in 2008 and ended the day of the last HbA_1c_ measurement in 2017, or the day when the events (e.g., renal or other complications) were first identified. Individuals without an event until the end of the follow-up were considered as censoring. Kaplan-Meier methods with log rank tests were used to estimate survivals of CKD and ESRD according to baseline or dynamic HbA_1c_ groups. An extended Cox model was used to identify risk factors of the events. We considered the dynamic HbA_1c_ value as a time-varying covariate. Statistically significant variables in the univariable Cox model were included in the multivariable Cox model. Variable selection was performed through a backward stepwise process until the smallest Akaike information criterion value had been reached. Hazard ratios (HRs) of the baseline and dynamic HbA_1c_ group were presented with 95% confidence intervals, after adjustment for age, sex, BMI, systolic blood pressure, HDL cholesterol, antihypertensive, glucose-lowering agents, baseline proteinuria, and eGFR. In addition, we performed a Cox regression with propensity scores as weights for the sex ratio for each HbA_1c_ group (baseline and dynamic). The proportional hazard assumption was evaluated using the Schoenfeld residuals method. When the proportional hazard assumption was violated, the time-dependent coefficient analysis was considered, and the hazard ratios were estimated separately in appropriate time intervals. Data analyses were performed by a statistical expert (Y.L.) using the R Statistical Package, Version 3.5.1 (R Foundation for Statistical Computing, Vienna, Austria). Statistical significance was set at *p* < 0.05.

## 3. Results

### 3.1. Patient Characteristics according to Baseline HbA_1c_

The patients' baseline characteristics are shown in Table 1. The mean age was 64.67 ± 6.60 years, and 85.48% were men. The longitudinal data of median HbA_1c_ are presented every two years according to the HbA_1c_ category in Electronic Supplementary Figure 1. The HbA_1c_ curve maintained similar values in each group over time, except for the initial values at a higher HbA_1c_ ≥ 64 mmol/mol (8.0%), which shows a marked decrease afterwards.

#### 3.1.1. Renal Complications

**Figure 1:**
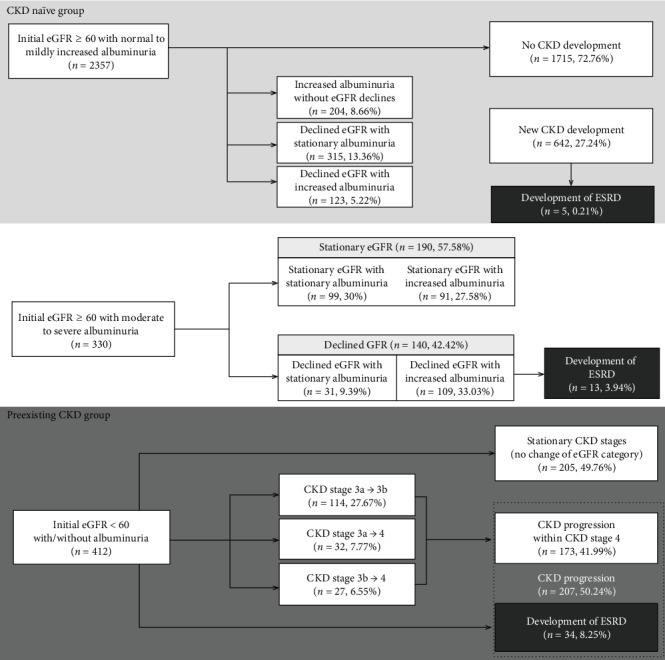
Development of chronic kidney disease (CKD) in the CKD-naïve group and CKD progression in the preexisting CKD group after 10 years. Abbreviations: CKD: chronic kidney disease; ESRD: end-stage renal disease; eGFR: estimated glomerular filtration rate.

At the beginning of the study, 2357 (76.06%) patients were CKD-naïve and 412 (23.94%) patients had preexisting CKD (Figure 1). During the study period, 27.24% of the CKD-naïve patients developed new CKD; 50.24% of the preexisting CKD patients had CKD progression. In the groups with initial eGFR ≥ 60 mL/min/1.73 m^2^ with moderate to severe albuminuria, 42.42% of the patients progressed to declined eGFR. The group with initial eGFR < 60 mL/min/1.73 m^2^ with/without albuminuria developed ESRD at the highest rate of 8.25%, while the overall incidence of ESRD was 1.7%.

#### 3.1.2. Hazard Ratio of Glycemic Control for CKD and ESRD

**Figure 2:**
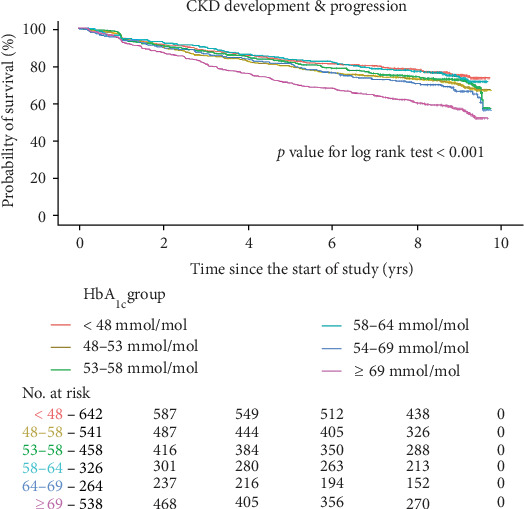
Kaplan-Meier curves for the development or progression of chronic kidney disease according to baseline glycated hemoglobin. Abbreviations: HbA_1c_: glycated hemoglobin; CKD: chronic kidney disease; No.: number. Kaplan-Meier curves stratified by baseline HbA_1c_ into six groups. Renal outcome was defined as a composite event of the first CKD development in the CKD-naïve group (*n* = 2357) and CKD progression in the preexisting CKD group (*n* = 412).

**Figure 3:**
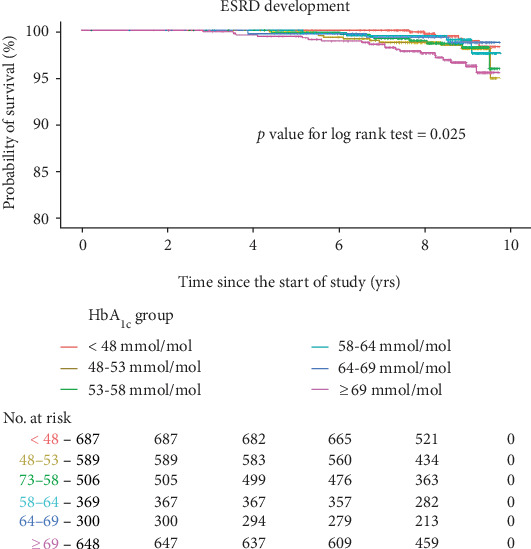
Kaplan-Meier curves for the development of end-stage renal disease according to baseline glycated hemoglobin. Abbreviations: HbA_1c_: glycated hemoglobin; ESRD: end-stage renal disease; No.: number. Kaplan-Meier curves stratified by baseline HbA_1c_ into six groups. Renal outcome was defined as the first ESRD development in all subjects including CKD naïve (*n* = 2357), normal GFR with albuminuria (*n* = 330), and preexisting CKD (*n* = 412).

Figures 2 and 3 present survival rates for the first progression of CKD or ESRD according to baseline HbA_1c_. The group with HbA_1c_ ≥ 64 mmol/mol (8.0%) had a higher progression rate for CKD (log rank *p* < 0.001 for both baseline and dynamic HbA_1c_, Figure 2, and Supplementary Figure 2). For ESRD development, the group with HbA_1c_ ≥ 69 mmol/mol (8.5%) had a higher risk compared to the rest of the groups (log rank *p* = 0.025 for baseline HbA_1c_ in Figure 3, and log rank *p* = 0.003 for dynamic HbA_1c_ in the Supplementary Figure 3).

In the multiple Cox regression (Table 2), baseline HbA1c showed high time-dependent hazard ratios for CKD from the second year of the study and ESRD for the entire follow-up period. Dynamic HbA_1c_ was also a risk factor for CKD with the satisfaction of proportional hazard assumption, but not for ESRD (Supplementary Table 1).

For the group with baseline HbA_1c_ ≥ 69 mmol/mol (8.5%), the hazard ratio was 1.98 (95% CI, 1.52–2.57) for CKD development or progression and 4.52 (95% CI, 1.44–14.13) for ESRD development (Table 3). For dynamic HbA_1c_, there was an increased risk for CKD at ≥69 mmol/mol (8.5%, HR = 2.32; 95% CI, 1.76–3.05, Supplementary Table 2). The composite risk for CKD was marginally elevated in patients with baseline HbA_1c_ 64–69 mmol/mol (8.0–8.5%) even after weighted analysis (Table 3, Supplementary Table 2-4). In contrast, there was no increased risk in those with baseline or dynamic HbA_1c_ of <64 mmol/mol.

### 3.2. Other Microvascular Complications

Both baseline and dynamic HbA_1c_ were associated with neuropathy risk at 0-60 months, retinopathy risk after 60 (70 for dynamic) months, and risk for diabetic foot over the entire period (Table 2 and Supplementary Table 1). The HbA_1c_ group with ≥58 mmol/mol (7.5%) showed a higher risk for neuropathy (during 22–110 months) and retinopathy (after 60 months, Table 3 and Supplementary Table 2–4) before and after weighted analysis. Meanwhile, there was no increased risk for any complications at baseline or dynamic HbA_1c_ < 58 mmol/mol (7.5%) for the entire period.

## 4. Discussion

The current study showed the development and progression of renal complications in the Korean Veterans Diabetes Cohort, which is mostly composed of older men with type 2 diabetes. A higher HbA_1c_ ≥ 69 mmol/mol (8.5%) was associated with an increased risk of renal complications, while the risk for other complications was elevated at a HbA_1c_ of ≥58 mmol/mol (7.5%) with a time-varying effect. Based on our 10-year analysis, a slight upward adjustment of target HbA_1c_ below 58 mmol/mol (7.5%) can be acceptable in older patients to reduce the risk for renal and other complications for a decade or so.

This is somewhat higher than the usual HbA_1c_ target of 48–53 mmol/mol (6.5–7.0%). A meta-analysis of UKPDS, Action in Diabetes and Vascular Disease (ADVANCE), and ACCORD trials showed that intensive glycemic control (median HbA_1c_: 46–57 mmol/mol, 6.4–7.4%) reduced the risk for the development of CKD compared to that of a conventional control (median HbA_1c_: 56–79 mmol/mol, 7.3–9.4%) [19]. Many guidelines still recommend maintaining a target HbA_1c_<48 mmol/mol (6.5%) or 53 mmol/mol (7.0%). Only a few guidelines suggested the targets for HbA_1c_ below 58 mmol/mol (7.5%), 64 mmol/mol (8.0%), or 69 mmol/mol (8.5%) in elderly type 2 diabetes patients.

In elderly patients, the correlation between glycemic control and renal outcomes tends to be more ambiguous with their comorbid condition as shown in a previous large-scale cohort study [20]. In a posttrial follow-up of the ADVANCE trial, the benefit of intensive glucose control on ESRD was only in the groups with no CKD or CKD stage 1–2, but not significant in the group with CKD stage 3 [21]. This is relevant to the current patients who were elderly veterans with several comorbidities. For elderly patients, it may be reasonable to suggest a slightly higher glycemic control target, because previous studies showed higher mortality at HbA_1c_ < 42 mmol/mol (6.0%), with the relationship showing a U-shaped curve [10, 20, 22–26]. Some studies have suggested individualized strategies for diabetes treatment, especially in the elderly [27]. This is consistent with the purpose and the results of our study, suggesting a slightly higher target HbA_1c_ while minimizing the risk of long-term complications.

Renal outcomes are variably defined as a composite of deterioration in albuminuria and GFR decline, which may lead to discrepancies in the study results. In UKPDS, ACCORD, ADVANCE, and VADT, intensive glycemic control reduced the risk of albuminuria, but not the risk of GFR decline [4, 7–9, 14]. The lower risk in renal outcome at somewhat higher HbA_1c_ of ≥69 mmol/mol (8.5%) in this study might be because our definition of CKD included decreased eGFR, rather than only incident albuminuria according to the recent KDIGO guideline [18].

Blood pressure control, together with antihypertensive drugs, is effective in preventing CKD progression [28–30]. In our cohort, angiotensin blockers had already been administered in most patients with hypertension. Thus, the effect of hypertension is presumed to be diluted. Recent studies demonstrated that sodium-glucose cotransporter 2 or glucagon-like peptide-1 also have protective effects against CKD progression [31, 32]. Because these drugs were not introduced before 2017, they were not included in our study.

There are only a few studies on the progression of renal outcome in type 2 diabetes. Data from UKPKDS showed the prevalence of diabetic kidney disease of 28.6% and renal replacement therapy of 0.4% at 10 years [33], consistent with our findings. A recent study reported no difference in the type of diabetes, age, and sex in the progression of nephropathy [34]. Considering this, the findings from our cohort, which is comprised of mostly older men, may be applied to younger patients or women.

This study has some limitations. Because of the retrospective cohort design, there may be unmeasured confounding factors, and a causal relationship cannot be made. There were some missing data on smoking or diabetes duration, which are known to be important risk factors for renal outcomes [35]. Earlier studies, however, failed to find significant associations between renal complications and smoking [14, 36, 37] or diabetes duration [10]. Because the current study sample consists primarily of male veterans, results must be carefully interpreted when attempting to generalize the findings to the entire population, although our weighted analysis with the sex ratio showed similar results. In our study, neuropathy and retinopathy, as well as baseline comorbidities, were identified using the diagnostic code, and there may be a risk of overestimation or misclassification, and variations depending on the clinicians. In addition, there may be selection bias, because cases with missing data for HbA_1c_, eGFR, and proteinuria at the beginning and end of the study were excluded. However, a major strength of the present study is the large veteran cohort of elderly men with a long-term follow-up observation of 10 years and which features a low dropout rate. The natural history of diabetic kidney disease, which remains to be clarified to date, was also reviewed in these patients. Moreover, because clinical decisions are often based on the values at one point of time, rather than integrated values, the analysis of baseline HbA_1c_ may be easier to interpret in the real world.

## 5. Conclusions

The progression of renal complications was analyzed according to baseline or dynamic HbA_1c_ for a decade. Although careful interpretation is needed, a less stringent glycemic target is acceptable to prevent renal and other complications. Our findings will be valuable for establishing optimal glycemic targets, particularly in elderly patients with a long duration of diabetes and comorbidities.

## Figures and Tables

**Table 1:** Baseline patient characteristics according to the glycosylated hemoglobin categories.

	Baseline HbA_1c_							
	Overall	<48 mmol/mol	48-53 mmol/mol	53-58 mmol/mol	58-64 mmol/mol	64-69 mmol/mol	≥69 mmol/mol	
		<6.5%	6.5–7%	7–7.5%	7.5–8%	8–8.5%	≥8.5%	
	(*n* = 3099)	(*n* = 687)	(*n* = 589)	(*n* = 506)	(*n* = 369)	(*n* = 300)	(*n* = 648)	*p* ^∗^
Age (years)	64.67 ± 6.60	65.10 ± 6.92	65.16 ± 6.22	65.25 ± 6.34	64.48 ± 6.88	64.93 ± 5.91	63.29 ± 6.77	<0.001
Sex, male *n* (%)	2649 (85.48)	631 (91.85)	502 (85.23)	429 (84.78)	313 (84.82)	250 (83.33)	524 (80.86)	<0.001
Body mass index (kg/m^2^)	25.04 ± 3.20	24.78 ± 3.13	24.97 ± 3.28	25.26 ± 2.94	25.02 ± 3.12	25.27 ± 3.72	25.07 ± 3.19	0.176
HbA_1c_ (mmol/mol)	58.68 ± 16.07	41.74 ± 3.94	49.72 ± 1.53	55.07 ± 1.53	60.65 ± 1.53	66.00 ± 1.53	83.27 ± 13.77	<0.001
HbA_1c_ (%)	7.52 ± 1.47	5.97 ± 0.36	6.70 ± 0.14	7.19 ± 0.14	7.70 ± 0.14	8.19 ± 0.14	9.77 ± 1.26	<0.001
SBP (mmHg)	129.11 ± 16.93	128.52 ± 17.50	128.95 ± 16.40	130.00 ± 17.10	127.66 ± 15.36	130.79 ± 17.19	129.23 ± 17.35	0.449
DBP (mmHg)	74.14 ± 10.15	74.25 ± 10.12	73.71 ± 9.34	74.25 ± 10.32	72.97 ± 10.08	74.02 ± 10.28	74.96 ± 10.60	0.268
HDL cholesterol (mmol/L) T_a_:	1.18 ± 0.34	1.22 ± 0.34 a	1.19 ± 0.36 a	1.19 ± 0.35 ab	1.16 ± 0.35 ab	1.19 ± 0.32 ab	1.13 ± 0.31 b	<0.001
LDL cholesterol (mmol/L)	2.61 ± 0.76	2.56 ± 0.76	2.63 ± 0.74	2.57 ± 0.76	2.67 ± 0.68	2.59 ± 0.78	2.67 ± 0.80	0.016
Triglyceride (mmol/L) T_a_:	4.17 ± 3.30	4.00 ± 3.16 b	4.04 ± 2.92 ab	3.92 ± 2.35 b	4.19 ± 2.54 ab	4.34 ± 3.44 ab	4.56 ± 4.45 a	0.001
Creatinine (*μ*mol/L) T_a_:	87.52 ± 23.87	88.40 ± 22.10 ab	90.17 ± 27.40 a	87.52 ± 22.98 ab	85.75 ± 22.10 ab	85.75 ± 21.22 ab	84.86 ± 23.87 b	0.001
Uric acid (*μ*mol/L) T_a_:	312.30 ± 86.25	324.19 ± 87.44 a	317.65 ± 91.01 ab	311.11 ± 82.68 ab	308.73 ± 81.49 ab	300.40 ± 81.49 b	302.18 ± 86.85 b	<0.001
Albuminuria, *n* (%)								<0.001
Normal to mildly increased	2634 (85.00)	614 (89.37)	509 (86.42)	445 (87.94)	312 (84.55)	250 (83.33)	504 (77.78)	
Moderately increased	411 (13.26)	68 (9.90)	71 (12.05)	58 (11.46)	52 (14.09)	44 (14.67)	118 (18.21)	
Severely increased	54 (1.74)	5 (0.73)	9 (1.53)	3 (0.59)	5 (1.36)	6 (2.00)	26 (4.01)	
Stage of CKD, *n* (%)								0.633
Stage 1–2 (eGFR ≥ 60 mL/min/1.73 m^2^)	2687 (86.71)	595 (86.61)	495 (84.04)	446 (88.14)	326 (88.35)	260 (86.67)	565 (87.19)	
Stage 3 (eGFR 30–60 mL/min/1.73 m^2^)	400 (12.90)	90 (13.10)	90 (15.28)	58 (11.46)	41 (11.11)	40 (13.33)	81 (12.50)	
Stage 4 (eGFR 15–30 mL/min/1.73 m^2^)	12 (0.39)	2 (0.29)	4 (0.68)	2 (0.40)	2 (0.54)	0 (0.0)	2 (0.31)	
Comorbidities								
Hypertension, *n* (%)	2593 (83.67)	573 (83.41)	486 (82.51)	425 (83.99)	298 (80.76)	247 (82.33)	564 (87.04)	0.121
Hypercholesterolemia, *n* (%)	2474 (79.83)	532 (77.44)	475 (80.65)	390 (77.08)	299 (81.03)	244 (81.33)	534 (82.41)	0.131
Coronary heart disease, *n* (%)	1509 (48.69)	349 (50.80)	287 (48.73)	237 (46.84)	167 (45.26)	138 (46.00)	331 (51.08)	0.305
Congestive heart failure, *n* (%)	380 (12.26)	92 (13.39)	67 (11.38)	53 (10.47)	39 (10.57)	43 (14.33)	86 (13.27)	0.353
Cerebrovascular disease, *n* (%)	697 (22.49)	171 (24.89)	128 (21.73)	121 (23.91)	75 (20.33)	62 (20.67)	140 (21.60)	0.427
Medication								
Statin, *n* (%)	1576 (50.86)	329 (47.89)	303 (51.44)	251 (49.60)	202 (54.74)	158 (52.67)	333 (51.39)	0.356
Antihypertensive								
ACE inhibitor/ARB, *n* (%)	1826 (58.92)	398 (57.93)	346 (58.74)	297 (58.70)	206 (55.83)	181 (60.33)	398 (61.42)	0.597
Beta blocker, *n* (%)	668 (21.56)	157 (22.85)	145 (24.62)	98 (19.37)	77 (20.87)	61 (20.33)	130 (20.06)	0.252
Calcium channel blocker, *n* (%)	986 (31.82)	259 (37.70)	192 (32.60)	168 (33.20)	103 (27.91)	90 (30.00)	174 (26.85)	0.001
Diuretics, *n* (%)	976 (31.49)	232 (33.77)	187 (31.75)	151 (29.84)	115 (31.17)	86 (28.67)	205 (31.64)	0.632
Glucose-lowering drugs								
Insulin, *n* (%)	401 (12.94)	29 (4.22)	51 (8.66)	60 (11.86)	41 (11.11)	55 (18.33)	165 (25.46)	<0.001
Metformin, *n* (%)	2265 (73.09)	486 (70.74)	402 (68.25)	360 (71.15)	284 (76.96)	222 (74.00)	511 (78.86)	<0.001
Sulfonylurea, *n* (%)	2224 (71.77)	484 (70.45)	392 (66.55)	382 (75.49)	293 (79.40)	226 (75.33)	447 (68.98)	<0.001
Alpha-glucosidase inhibitor (voglibose), *n* (%)	440 (14.20)	94 (13.68)	65 (11.04)	66 (13.04)	67 (18.16)	43 (14.33)	105 (16.20)	0.029
Thiazolidinedione (actos), *n* (%)	249 (8.03)	45 (6.55)	47 (7.98)	43 (8.50)	33 (8.94)	25 (8.33)	56 (8.64)	0.705
Nateglinide (fastic), *n* (%)	132 (4.26)	34 (4.95)	40 (6.79)	15 (2.96)	18 (4.88)	9 (3.00)	16 (2.47)	0.002

^∗^ *p* value: categorical data were analyzed using Fisher's test or the Chi-square test; continuous data were analyzed using ANOVA; if *p* < 0.05 in ANOVA, post hoc analysis was performed using Tukey's test. T_a_: the same letters indicate nonspecific difference between groups based on Tukey's test. Abbreviations: HbA_1c_: glycated hemoglobin; eGFR: estimated glomerular filtration rate; SBP: systolic blood pressure; DBP: diastolic blood pressure; CKD: chronic kidney disease; ACE: angiotensin-converting enzyme; LDL: low-density lipoprotein; HDL: high-density lipoprotein.

**Table 2:** Cox models of baseline glycated hemoglobin for the risk of renal and other microvascular complications.

Risk factors	CKD development & progression	ESRD development	Retinopathy development	Neuropathy development	Diabetic foot development
Simple Cox regression					
HbA_1c_, per 1% (11 mmol/mol)	1.19 (1.14–1.24)^∗^	1.33 (1.16–1.52)^∗^			
Time < 1800 days			1.00 (0.94–1.06)	1.19 (1.11–1.27)^∗^	1.19 (0.91–1.55)
Time ≥ 1800 days			1.22 (1.15–1.29)^∗^	1.08 (0.97–1.19)	1.24 (1.09–1.40)^∗^
Age, per 2 years	1.09 (1.07–1.12)^∗^	0.95 (0.88–1.03)	0.98 (0.96–1)	0.99 (0.97–1.02)	0.99 (0.93–1.06)
Sex, female vs. male		1.44 (0.57–3.63)	1.26 (1.02–1.57)^†^	1.24 (0.95–1.62)	3.01 (1.10–8.23^)†^
Time < 900 days	1.30 (0.93–1.82)				
Time ≥ 900 days	0.68 (0.55–0.83)^∗^				
BMI, per 2 kg/m^2^	0.99 (0.94–1.04)	1.02 (0.86–1.21)	1.02 (0.97–1.08)	0.95 (0.89–1.01)	0.94 (0.82–1.08)
SBP, per 10 mmHg	1.05 (1.00–1.1)^†^	1.21 (1.04–1.41)^†^		0.96 (0.90–1.02)	0.99 (0.88–1.11)
Time < 1800 days			0.92 (0.86–0.98)^†^		
Time ≥ 1800 days			1.04 (0.97–1.11)		
DBP, per 10 mmHg	1.00 (0.93–1.08)		1.01 (0.93–1.09)	0.95 (0.86–1.05)	1.15 (0.94–1.4)
Time < 3000 days		1.39 (0.99–1.92)			
Time ≥ 3000 days		0.70 (0.45–1.08)			
LDL cholesterol, per 0.13 mmol/L	0.99 (0.98–1)	0.93 (0.89–0.98)^∗^	0.99 (0.98–1)	1.00 (0.98–1.01)	0.98 (0.94–1.01)
HDL cholesterol, per 0.13 mmol/L		0.86 (0.75–0.98)^†^	1.00 (0.97–1.02)	0.97 (0.93–1)	0.79 (0.71–0.88)^∗^
Time < 2400 days	0.90 (0.86–0.93)^∗^				
Time ≥ 2400 days	0.97 (0.91–1.03)				
Triglyceride, per 0.52 mmol/L	1.01 (1.00–1.02)^†^	1.02 (0.99–1.04)	1.00 (0.99–1.01)	1.00 (0.98–1.02)	1.02 (1.00–1.04)
Uric acid, per 59.48 *μ*mol/L	1.30 (1.24–1.36)^∗^	1.63 (1.40–1.9)^∗^	1.03 (0.98–1.09)	0.95 (0.89–1.02)	1.33 (1.16–1.53)^∗^
Statin		1.48 (0.85–2.58)	1.12 (0.98–1.29)	1.14 (0.95–1.37)	1.19 (0.78–1.82)
Time < 2400 days	1.21 (1.04–1.40)^†^				
Time ≥ 2400 days	0.78 (0.58–1.06)				
Antihypertensive agent		5.97 (1.86–19.15)^∗^	1.27 (1.08–1.5)^∗^		1.63 (0.97–2.74)
Time < 2400 days	2.18 (1.78–2.66)^∗^			1.62 (1.25–2.11)^∗^	
Time ≥ 2400 days	1.20 (0.86–1.67)			0.96 (0.66–1.40)	
Glucose-lowering agent	1.25 (0.76–2.04)	1.12 (0.16–8.15)	1.07 (0.63–1.81)	2.71 (1.01–7.24)^†^	1.48 (0.21–10.67)
eGFR		0.93 (0.92–0.95)^∗^	1.00 (1.00–1.00)	1.00 (0.99–1.00)	
Time < 2400 days	0.97 (0.96–0.97)^∗^				
Time ≥ 2400 days	0.99 (0.98–1.00)^†^				
Proteinuria, + vs (ref = negative or trace)	3.33 (2.67–4.14)^∗^	21.64 (11.12–42.09)^∗^	1.44 (1.21–1.72)^∗^	1.04 (0.80–1.35)	
Time < 3650 days					2.20 (1.34–3.60)^∗^
Time ≥ 3650 days					0.00 (0.00–Inf)
Multiple Cox regression					
HbA_1c_, per 1% (11 mmol/mol)		1.37 (1.17–1.60)^∗^			1.20 (1.06–1.35)^∗^
Time < 365 days	1.08 (0.96–1.22)				
Time ≥ 365 days	1.21 (1.15–1.28)^∗^				
Time < 1800 days			0.98 (0.91–1.05)	1.18 (1.11–1.26)^∗^	
Time ≥ 1800 days			1.18 (1.11–1.26)^∗^	1.07 (0.97–1.18)	
Age, per 2 years	1.06 (1.03–1.09)^∗^				
Sex, female vs. male					3.29 (0.79–13.63)
SBP, per 10 mmHg	1.04 (0.99–1.10)	1.24 (1.04–1.48)^†^			
Time < 1800 days			0.91 (0.86–0.97)^∗^		
Time ≥ 1800 days			1.04 (0.97–1.11)		
LDL cholesterol, per 0.13 mmol/L		0.93 (0.88–0.98)^∗^			
HDL cholesterol, per 0.13 mmol/L	0.94 (0.90–0.98)^∗^				0.82 (0.73–0.93)^∗^
Uric acid, per 59.48 *μ*mol/L	1.15 (1.08–1.22)^∗^				1.23 (1.06–1.44)^∗^
Antihypertensive agent	1.29 (1.03–1.63)^†^				
Time < 1800 days			1.38 (1.05–1.81)^†^	1.62 (1.22–2.15)^∗^	
Time ≥ 1800 days			0.90 (0.69–1.18)	1.11 (0.80–1.54)	
Glucose-lowering agent				2.42 (0.90–6.47)	
eGFR		0.94 (0.93–0.96)^∗^			
Time < 365 days	0.97 (0.96–0.98)^∗^				
Time ≥ 365 days	0.99 (0.98–0.99)^∗^				
Proteinuria, + vs (ref = negative or trace)		19.61 (7.54–51.03)^∗^	1.31 (1.08–1.58)^∗^		

A composite analysis of CKD development and progression was performed in CKD naïve and preexisting CKD groups (*n* = 2769). Analysis for the development of ESRD was performed in all subjects (*n* = 3099). Data are presented as hazard ratio (95% confidential interval). ^∗^*p* < 0.01, ^†^*p* < 0.05. Abbreviations: HbA_1c_: glycated hemoglobin; BMI: body mass index; SBP: systolic blood pressure; DBP: diastolic blood pressure; LDL: low-density lipoprotein; HDL: high-density lipoprotein; eGFR: estimated glomerular filtration rate.

**Table 3:** Cox models for the risk of renal and other microvascular complications according to the baseline glycated hemoglobin level.

Outcome	Model	Baseline HbA_1c_					
Renal complications		<48 mmol/mol	48-53 mmol/mol	53-58 mmol/mol	58-64 mmol/mol	64-69 mmol/mol	≥69 mmol/mol
		<6.5%	6.5-7%	7–7.5%	7.5–8%	8–8.5%	≥8.5%
CKD development or progression	HR (95% CI)	1	1.29 (0.96-1.72)	1.17 (0.86-1.59)	1.07 (0.76-1.49)	1.42 (1.00-2.01)	1.98 (1.52-2.57)^∗^
Development of CKD		1	1.21 (0.86-1.70)	1.08 (0.76-1.54)	1.10 (0.74-1.63)	1.33 (0.88-2.02)	1.86 (1.38-2.52)^∗^
Progression of CKD		1	1.57 (0.90-2.72)	1.36 (0.72-2.59)	1.12 (0.58-2.14)	1.66 (0.85-3.22)	2.18 (1.27-3.75)^∗^
ESRD development		1	2.76 (0.80-9.55)	1.92 (0.53-6.89)	1.86 (0.48-7.11)	1.27 (0.23-7.14)	4.52 (1.44-14.13)^∗^
*Other microvascular complications*							
Development of retinopathy							
Time < 1800 days		1	1.05 (0.73-1.53)	1.05 (0.72-1.55)	0.75 (0.48-1.18)	0.92 (0.57-1.47)	1.02 (0.72-1.44)
Time ≥ 1800 days		1	1.37 (0.80-2.33)	1.58 (0.93-2.67)	2.31 (1.39-3.86)^∗^	3.34 (2.01-5.56)^∗^	2.83 (1.81-4.42)^∗^
Neuropathy							
Time < 660 days		1	1.26 (0.61-2.58)	0.79 (0.34-1.83)	1.01 (0.42-2.40)	1.18 (0.47-2.92)	2.09 (1.12-3.92)^†^
660 ≤ time < 3300 days		1	1.14 (0.69-1.88)	0.91 (0.53-1.56)	2.15 (1.33-3.50)^∗^	1.84 (1.07-3.17)^†^	1.94 (1.25-3.00)^∗^
Time ≥ 3300 days		1	0.40 (0.11-1.47)	0.88 (0.27-2.84)	0.52 (0.06-4.29)	1.45 (0.36-5.73)	0.47 (0.14-1.58)
Diabetic foot		1	0.40 (0.14-1.11)	0.31 (0.10-0.98)^†^	1.25 (0.57-2.75)	0.41 (0.12-1.48)	1.35 (0.69-2.65)

A composite analysis of CKD development and progression was performed in CKD-naïve and preexisting CKD groups (*n* = 2769). Analysis for the development of ESRD was performed in all subjects (*n* = 3099). Data are shown as HR (95% confidence interval). ^∗^*p* < 0.01, ^†^*p* < 0.05, adjusted for age, sex, body mass index, systolic blood pressure, high-density lipoprotein cholesterol, antihypertensive, and glucose-lowering agents. Abbreviations: CKD: chronic kidney disease; ESRD: end-stage renal disease; HR: hazard ratio; CI: confidence interval.

## Data Availability

The datasets generated and analyzed during the current study are available from the corresponding author on reasonable request.

## Abbreviations

ACCORD: Action to Control Cardiovascular Risk in Diabetes
ADVANCE: Action in Diabetes and Vascular Disease
BMI: Body mass index
CKD: Chronic kidney disease
eGFR: Estimated glomerular filtration rate
ESRD: End-stage renal disease
HDL: High-density lipoprotein
KDIGO: Kidney Disease: Improving Global Outcomes
LDL: Low-density lipoprotein
UKPDS: UK Prospective Diabetes Study
VADT: Veterans Affairs Diabetes Trial.

## Supplementary Materials

Supplementary Materials . Figure S1-3 in the Supplementary Material for comprehensive image analysis (changes in baseline glycated hemoglobin, Kaplan-Meier curves for dynamic glycated hemoglobin). Table S1-4 in the Supplementary Material for a statistical complement for dynamic HbA1c or propensity score-weighted analysis.

## References

[1] The International Expert Committee (2009). International expert committee report on the role of the A1C assay in the diagnosis of diabetes. *Diabetes Care*.

[2] American Diabetes Association (2013). Diagnosis and classification of diabetes mellitus. *Diabetes Care*.

[3] Ogurtsova K., da Rocha Fernandes J. D., Huang Y. (2017). IDF Diabetes Atlas: global estimates for the prevalence of diabetes for 2015 and 2040. *Diabetes Research and Clinical Practice*.

[4] UK Prospective Diabetes Study (UKPDS) Group (1998). Intensive blood-glucose control with sulphonylureas or insulin compared with conventional treatment and risk of complications in patients with type 2 diabetes (UKPDS 33). *The Lancet*.

[5] American Diabetes A. (2018). 6. Glycemic Targets:Standards of medical care in diabetes-2019. *Diabetes Care*.

[6] The Diabetes Control and Complications Trial Research Group (1993). The effect of intensive treatment of diabetes on the development and progression of long-term complications in insulin-dependent diabetes mellitus. *The New England Journal of Medicine*.

[7] Duckworth W., Abraira C., Moritz T. (2009). Glucose control and vascular complications in veterans with type 2 diabetes. *The New England Journal of Medicine*.

[8] The ADVANCE Collaborative Group (2008). Intensive blood glucose control and vascular outcomes in patients with type 2 diabetes. *The New England Journal of Medicine*.

[9] The Action to Control Cardiovascular Risk in Diabetes Study Group (2008). Effects of intensive glucose lowering in type 2 diabetes. *The New England Journal of Medicine*.

[10] Huang E. S., Liu J. Y., Moffet H. H., John P. M., Karter A. J. (2011). Glycemic control, complications, and death in older diabetic patients: the diabetes and aging study. *Diabetes Care*.

[11] Currie C. J., Peters J. R., Tynan A. (2010). Survival as a function of HbA_1c_ in people with type 2 diabetes: a retrospective cohort study. *The Lancet*.

[12] Stratton I. M., Adler A. I., Neil H. A. (2000). Association of glycaemia with macrovascular and microvascular complications of type 2 diabetes (UKPDS 35): prospective observational study. *BMJ*.

[13] Jha V., Garcia-Garcia G., Iseki K. (2013). Chronic kidney disease: global dimension and perspectives. *The Lancet*.

[14] Agrawal L., Azad N., Emanuele N. V. (2011). Observation on renal outcomes in the Veterans Affairs Diabetes Trial. *Diabetes Care*.

[15] Zoungas S., Arima H., Gerstein H. C. (2017). Effects of intensive glucose control on microvascular outcomes in patients with type 2 diabetes: a meta-analysis of individual participant data from randomised controlled trials. *The Lancet Diabetes and Endocrinology*.

[16] Tonelli M., Muntner P., Lloyd A. (2012). Risk of coronary events in people with chronic kidney disease compared with those with diabetes: a population-level cohort study. *The Lancet*.

[17] Levey A. S., Coresh J., Greene T. (2006). Using standardized serum creatinine values in the modification of diet in renal disease study equation for estimating glomerular filtration rate. *Annals of Internal Medicine*.

[18] Inker L. A., Astor B. C., Fox C. H. (2014). KDOQI US commentary on the 2012 KDIGO clinical practice guideline for the evaluation and management of CKD. *American Journal of Kidney Diseases*.

[19] Coca S. G., Ismail-Beigi F., Haq N., Krumholz H. M., Parikh C. R. (2012). Role of intensive glucose control in development of renal end points in type 2 diabetes mellitus: systematic review and meta-analysis intensive glucose control in type 2 diabetes. *Archives of Internal Medicine*.

[20] Shurraw S., Hemmelgarn B., Lin M. (2011). Association between glycemic control and adverse outcomes in people with diabetes mellitus and chronic kidney disease: a population-based cohort study. *Archives of Internal Medicine*.

[21] Zoungas S., Chalmers J., Neal B. (2014). Follow-up of blood-pressure lowering and glucose control in type 2 diabetes. *The New England Journal of Medicine*.

[22] Freedman B. I. (2012). A critical evaluation of glycated protein parameters in advanced nephropathy: a matter of life or death: time to dispense with the hemoglobin A1C in end-stage kidney disease. *Diabetes Care*.

[23] Ramirez S. P. B., McCullough K. P., Thumma J. R. (2012). Hemoglobin A(1c) levels and mortality in the diabetic hemodialysis population: findings from the Dialysis Outcomes and Practice Patterns Study (DOPPS). *Diabetes Care*.

[24] Riddle M. C., Ambrosius W. T., Brillon D. J. (2010). Epidemiologic relationships between A1C and all-cause mortality during a median 3.4-year follow-up of glycemic treatment in the ACCORD trial. *Diabetes Care*.

[25] Schernthaner G., Schernthaner-Reiter M. H. (2018). Diabetes in the older patient: heterogeneity requires individualisation of therapeutic strategies. *Diabetologia*.

[26] Kontopantelis E., Springate D. A., Reeves D. (2015). Glucose, blood pressure and cholesterol levels and their relationships to clinical outcomes in type 2 diabetes: a retrospective cohort study. *Diabetologia*.

[27] Nunes J. P. L., DeMarco J. P. (2019). A 7.0-7.7% value for glycated haemoglobin is better than a <7% value as an appropriate target for patient-centered drug treatment of type 2 diabetes mellitus. *Annals of Translational Medicine*.

[28] Tuttle K. R., Bakris G. L., Bilous R. W. (2014). Diabetic kidney disease: a report from an ADA Consensus Conference. *Diabetes Care*.

[29] James P. A., Oparil S., Carter B. L. (2014). 2014 evidence-based guideline for the management of high blood pressure in adults: report from the panel members appointed to the Eighth Joint National Committee (JNC 8). *JAMA*.

[30] Anderson R. J., Bahn G. D., Emanuele N. V., Marks J. B., Duckworth W. C., VADT Study Group (2014). Blood pressure and pulse pressure effects on renal outcomes in the Veterans Affairs Diabetes Trial (VADT). *Diabetes Care*.

[31] Wanner C., Inzucchi S. E., Lachin J. M. (2016). Empagliflozin and progression of kidney disease in type 2 diabetes. *The New England Journal of Medicine*.

[32] Mann J. F. E., Ørsted D. D., Brown-Frandsen K. (2017). Liraglutide and renal outcomes in type 2 diabetes. *The New England Journal of Medicine*.

[33] Adler A. I., Stevens R. J., Manley S. E. (2003). Development and progression of nephropathy in type 2 diabetes: the United Kingdom Prospective Diabetes Study (UKPDS 64). *Kidney International*.

[34] Hadjadj S., Cariou B., Fumeron F. (2016). Death, end-stage renal disease and renal function decline in patients with diabetic nephropathy in French cohorts of type 1 and type 2 diabetes. *Diabetologia*.

[35] Skupien J., Smiles A. M., Valo E. (2018). Variations in risk of end-stage renal disease and risk of mortality in an international study of patients with type 1 diabetes and advanced nephropathy. *Diabetes Care*.

[36] Marcovecchio M. L., Chiesa S. T., Armitage J. (2018). Renal and cardiovascular risk according to tertiles of urinary albumin-to-creatinine ratio: the adolescent type 1 diabetes cardio-renal intervention trial (AdDIT). *Diabetes Care*.

[37] Shankar A., Klein R., Klein B. E. K. (2006). The association among smoking, heavy drinking, and chronic kidney disease. *American Journal of Epidemiology*.

