# Supplementary material for: Renal Complication and Glycemic Control in Korean Veterans with Type 2 Diabetes: A 10-Year Retrospective Cohort Study
Source: J Diabetes Res. 2020 Jun 22;2020:9806790. doi: 10.1155/2020/9806790 (PMC7333055; doi:10.1155/2020/9806790)
Supplement: Supplementary Materials — . Figure S1-3 in the Supplementary Material for comprehensive image analysis (changes in baseline glycated hemoglobin, Kaplan-Meier curves for dynamic glycated hemoglobin). Table S1-4 in the Supplementary Material for a statistical complement for dynamic HbA1c or propensity score-weighted analysis. [file 9806790.f1.docx]

**Electronic Supplementary Tables**

| **Table S1. Cox Models for the Risk of Chronic Kidney Disease using Dynamic Glycated Hemoglobin level and Other Microvascular Complications** | | | | | |
| --- | --- | --- | --- | --- | --- |
| **Risk factors** | **CKD**  **Development & progression** | **ESRD**  **Development** | **Retinopathy**  **Development** | **Neuropathy**  **Development** | **Diabetic foot**  **Development** |
| **Simple Cox regression** |  |  |  |  |  |
| HbA_1c_, per 1% (11 mmol/mol) | 1.17 (1.12–1.22)* |  |  | 1.12 (1.05–1.19)* | 1.36 (1.21–1.53)* |
| time < 1800 days |  | 1.45 (1.01–2.10)† | 1.04 (0.97–1.12) |  |  |
| time ≥ 1800 days |  | 1.17 (0.97–1.41) | 1.23 (1.15–1.32)* |  |  |
| **Multiple Cox regression** |  |  |  |  |  |
| HbA_1c_, per 1% (11 mmol/mol) | 1.16 (1.10–1.22)* |  |  |  | 1.28 (1.12–1.46)* |
| time < 1800 days |  |  |  | 1.19 (1.11–1.28)* |  |
| time ≥ 1800 days |  |  |  | 0.97 (0.87–1.09) |  |
| time < 2100 days |  |  | 1.02 (0.95–1.09) |  |  |
| time ≥ 2100 days |  |  | 1.27 (1.18–1.37)* |  |  |
| Age, per 2 years | 1.05 (1.02–1.09)* |  |  |  |  |
| Sex, female vs. male |  |  |  |  | 3.24 (0.78–13.46) |
| SBP, per 10 mmHg | 1.05 (0.99–1.10) | 1.17 (0.99–1.39) |  |  |  |
| time < 2100 days |  |  | 0.93 (0.88–0.99)† |  |  |
| time ≥ 2100 days |  |  | 1.05 (0.98–1.13) |  |  |
| LDL cholesterol, per 0.13 mmol/L |  | 0.93 (0.88–0.99)† |  |  |  |
| HDL cholesterol, per 0.13 mmol/L | 0.94 (0.90–0.98)* |  |  |  | 0.84 (0.74–0.94)* |
| Uric acid, per 59.48 μmol/L | 1.14 (1.08–1.22)* |  |  |  | 1.25 (1.07–1.46)* |
| Antihypertensive agent | 1.27 (1.01–1.60)† |  |  |  |  |
| time < 1800 days |  |  |  | 1.63 (1.23–2.17)* |  |
| time ≥ 1800 days |  |  |  | 1.11 (0.80–1.55) |  |
| time < 2100 days |  |  | 1.26 (0.99–1.60) |  |  |
| time ≥ 2100 days |  |  | 0.92 (0.68–1.25) |  |  |
| Glucose-lowering agent |  |  |  | 2.52 (0.94–6.74) |  |
| eGFR |  | 0.95 (0.93–0.97)* |  |  |  |
| time < 1200 days | 0.98 (0.98–0.99)* |  |  |  |  |
| 1200 ≤ time < 2400 days | 0.98 (0.97–0.99)* |  |  |  |  |
| time ≥ 2400 days | 1.00 (1.00–1.00) |  |  |  |  |
| Proteinuria, + vs (ref=negative or trace) |  | 20.49 (7.84–53.59)* | 1.29 (1.06–1.56)† |  |  |

Data are presented as hazard ratio (95% confidential interval). A composite analysis of CKD development and progression was performed in CKD naïve and pre-existing CKD groups (n = 2769). Analysis for the development of ESRD was performed in all subjects (n = 3099). **P*<0.01, †*P*<0.05 Abbreviations: HbA_1c_, glycated hemoglobin; BMI, body mass index; SBP, systolic blood pressure; DBP, diastolic blood pressure; LDL, low-density lipoprotein; HDL, high-density lipoprotein; eGFR, estimated glomerular filtration rate

| **Table S2. Cox Models for the Risk of Chronic Kidney Disease and Other Microvascular Complications According to the Dynamic Glycated Hemoglobin Level** | | | | | | | |
| --- | --- | --- | --- | --- | --- | --- | --- |
| **Outcome** | **Model** | **Dynamic HbA_1c_** | | | | | |
| **Renal complications** |  | **<48 mmol/mol** | **48-53 mmol/mol** | **53-58 mmol/mol** | **58-64 mmol/mol** | **64-69 mmol/mol** | **≥69 mmol/mol** |
|  |  | **<6.5%** | **6.5-7%** | **7–7.5%** | **7.5–8%** | **8–8.5%** | **≥8.5%** |
| CKD development or progression | HR (95% CI) | 1 | 1.01 (0.76-1.35) | 0.99 (0.73-1.33) | 1.09 (0.79-1.51) | 1.20 (0.84-1.71) | 2.32 (1.76-3.05)* |
| Development of CKD |  | 1 | 0.96 (0.69-1.35) | 0.97 (0.68-1.36) | 1.05 (0.73-1.51) | 1.04 (0.67-1.61) | 2.46 (1.79-3.38)* |
| Progression of CKD |  | 1 | 1.19 (0.67-2.14) | 1.12 (0.60-2.07) | 1.09 (0.54-2.19) | 1.82 (0.94-3.52) | 2.07 (1.16-3.69)† |
| ESRD Development |  | 1 | 0.89 (0.27-2.88) | 1.80 (0.60-5.38) | 0.87 (0.22-3.41) | 0.80 (0.18-3.44) | 2.60 (0.90-7.51) |
| **Other microvascular complications** |  |  |  |  |  |  |  |
| Development of retinopathy |  |  |  |  |  |  |  |
| time < 1800 days |  | 1 | 0.89 (0.61-1.31) | 0.80 (0.54-1.18) | 1.09 (0.73-1.62) | 0.80 (0.50-1.27) | 1.14 (0.79-1.67) |
| time ≥ 1800 days |  | 1 | 0.82 (0.47-1.40) | 1.11 (0.67-1.81) | 1.77 (1.09-2.89)† | 2.74 (1.71-4.40)* | 2.66 (1.71-4.14)* |
| Neuropathy |  |  |  |  |  |  |  |
| time < 660 days |  | 1 | 1.00 (0.49-2.05) | 0.67 (0.30-1.52) | 0.72 (0.30-1.73) | 0.86 (0.34-2.13) | 2.72 (1.44-5.12)* |
| 660 ≤ time < 3300 days |  | 1 | 1.06 (0.65-1.73) | 0.91 (0.54-1.52) | 1.68 (1.03-2.72)† | 1.39 (0.81-2.38) | 2.23 (1.41-3.54)* |
| time ≥ 3300 days |  | 1 | 0.35 (0.10-1.18) | 0.30 (0.08-1.18) | 0.62 (0.17-2.31) | 0.29 (0.04-2.33) | 0.52 (0.15-1.74) |
| Diabetic foot |  | 1 | 0.99 (0.36-2.67) | 0.69 (0.24-2.02) | 1.02 (0.35-2.96) | 1.49 (0.53-4.16) | 3.26 (1.37-7.73)* |
| Data are presented as hazard ratio (95% confidential interval). A composite analysis of CKD development and progression was performed in CKD naïve group and pre-existing CKD group (n = 2769). Analysis for the development of ESRD was performed in total subjects (n = 3099). **p*<0.01, †*p*<0.05, adjusted for age, sex, body mass index, systolic blood pressure, high-density lipoprotein cholesterol, antihypertensive, and glucose-lowering agents  Abbreviations: CKD, chronic kidney disease; ESRD, end-stage renal disease; HR, hazards ratio; CI, confidence interval   \| **Table S3. Propensity Score Weighted Cox Models for the Risk of Chronic Kidney Disease and Other Microvascular Complications According to the Baseline Glycated Hemoglobin Level** \| \| \| \| \| \| \| \| \| --- \| --- \| --- \| --- \| --- \| --- \| --- \| --- \| \| **Outcome** \| **Model** \| **Baseline HbA_1c_** \| \| \| \| \| \| \| **Renal complications** \| **<48 mmol/mol** \| **48-53 mmol/mol** \| **53-58 mmol/mol** \| **58-64 mmol/mol** \| **64-69 mmol/mol** \| **≥69 mmol/mol** \| \| **<6.5%** \| **6.5-7%** \| **7–7.5%** \| **7.5–8%** \| **8–8.5%** \| **≥8.5%** \| \| CKD development or progression \| HR (95% CI) \| 1 \| 1.27 (0.94-1.71) \| 1.16 (0.85-1.59) \| 1.07 (0.76-1.50) \| 1.42 (1.00-2.02) \| 1.95 (1.48-2.57)* \| \| Development of CKD \|  \| 1 \| 1.20 (0.85-1.70) \| 1.07 (0.74-1.54) \| 1.11 (0.74-1.65) \| 1.36 (0.89-2.07) \| 1.83 (1.34-2.50)* \| \| Progression of CKD \|  \| 1 \| 1.58 (0.91-2.76) \| 1.37 (0.70-2.70) \| 1.09 (0.59-2.01) \| 1.69 (0.86-3.31) \| 2.18 (1.25-3.80)* \| \| ESRD Development \|  \| 1 \| 2.57 (0.75-8.76) \| 1.88 (0.46-7.74) \| 1.90 (0.52-6.97) \| 1.48 (0.25-8.84) \| 4.59 (1.50-14.02)* \| \| **Other microvascular complications** \|  \|  \|  \|  \|  \|  \|  \| \| Development of retinopathy \|  \|  \|  \|  \|  \|  \|  \| \| time < 1800 days \|  \| 1 \| 1.05 (0.72-1.53) \| 1.05 (0.71-1.55) \| 0.75 (0.48-1.18) \| 0.91 (0.57-1.46) \| 0.99 (0.70-1.41) \| \| time ≥ 1800 days \|  \| 1 \| 1.30 (0.76-2.24) \| 1.49 (0.87-2.55) \| 2.21 (1.31-3.72)* \| 3.18 (1.90-5.35)* \| 2.73 (1.72-4.35)* \| \| Neuropathy \|  \|  \|  \|  \|  \|  \|  \| \| time < 660 days \|  \| 1 \| 1.33 (0.65-2.74) \| 0.83 (0.36-1.92) \| 1.07 (0.45-2.58) \| 1.23 (0.49-3.07) \| 2.21 (1.17-4.16)† \| \| 660 ≤ time < 3300 days \|  \| 1 \| 1.18 (0.71-1.95) \| 0.93 (0.54-1.61) \| 2.24 (1.37-3.66)* \| 1.92 (1.12-3.31)† \| 1.97 (1.27-3.07)* \| \| time ≥ 3300 days \|  \| 1 \| 0.40 (0.12-1.33) \| 1.00 (0.29-3.47) \| 0.55 (0.11-2.78) \| 1.63 (0.42-6.29) \| 0.47 (0.16-1.38) \| \| Diabetic foot \|  \| 1 \| 0.43 (0.16-1.20) \| 0.42 (0.16-1.13) \| 1.37 (0.65-2.91) \| 0.45 (0.15-1.39) \| 1.41 (0.74-2.67) \| \| Data are presented as hazard ratio (95% confidential interval). A composite analysis of CKD development and progression was performed in CKD naïve and pre-existing CKD groups (n = 2769). Analysis for the development of ESRD was performed in all subjects (n = 3099). **p*<0.01, †*p*<0.05, adjusted for age, sex, body mass index, systolic blood pressure, high-density lipoprotein cholesterol, antihypertensive, and glucose-lowering agents  Abbreviations: CKD, chronic kidney disease; ESRD, end-stage renal disease; HR, hazards ratio; CI, confidence interval   \| **Table S4. Propensity Score Weighted Cox Models for the Risk of Chronic Kidney Disease and Other Microvascular Complications According to the Dynamic Glycated Hemoglobin Level** \| \| \| \| \| \| \| \| \| --- \| --- \| --- \| --- \| --- \| --- \| --- \| --- \| \| **Outcome** \| **Model** \| **Dynamic HbA_1c_** \| \| \| \| \| \| \| **Renal complications** \| **<48 mmol/mol** \| **48-53 mmol/mol** \| **53-58 mmol/mol** \| **58-64 mmol/mol** \| **64-69 mmol/mol** \| **≥69 mmol/mol** \| \| **<6.5%** \| **6.5-7%** \| **7–7.5%** \| **7.5–8%** \| **8–8.5%** \| **≥8.5%** \| \| CKD development or progression \| HR (95% CI) \| 1 \| 1.01 (0.75-1.36) \| 0.98 (0.72-1.34) \| 1.08 (0.77-1.51) \| 1.18 (0.82-1.71) \| 2.32 (1.73-3.10)* \| \| Development of CKD \|  \| 1 \| 0.95 (0.67-1.35) \| 0.95 (0.67-1.35) \| 1.03 (0.70-1.51) \| 1.03 (0.65-1.61) \| 2.47 (1.77-3.44)* \| \| Progression of CKD \|  \| 1 \| 1.29 (0.71-2.32) \| 1.14 (0.62-2.09) \| 1.18 (0.59-2.37) \| 1.92 (0.99-3.72) \| 2.20 (1.19-4.06)† \| \| ESRD Development \|  \| 1 \| 0.69 (0.20-2.38) \| 1.53 (0.50-4.66) \| 0.80 (0.21-3.02) \| 0.72 (0.17-3.08) \| 2.38 (0.85-6.64) \| \| **Other microvascular complications** \|  \|  \|  \|  \|  \|  \|  \| \| Development of retinopathy \|  \|  \|  \|  \|  \|  \|  \| \| time < 1800 days \|  \| 1 \| 0.89 (0.60-1.30) \| 0.80 (0.54-1.18) \| 1.08 (0.73-1.62) \| 0.78 (0.49-1.24) \| 1.13 (0.77-1.65) \| \| time ≥ 1800 days \|  \| 1 \| 0.81 (0.47-1.41) \| 1.10 (0.67-1.82) \| 1.76 (1.07-2.88)† \| 2.74 (1.69-4.45)* \| 2.68 (1.71-4.19)* \| \| Neuropathy \|  \|  \|  \|  \|  \|  \|  \| \| time < 660 days \|  \| 1 \| 1.05 (0.51-2.15) \| 0.68 (0.30-1.54) \| 0.76 (0.32-1.84) \| 0.88 (0.35-2.22) \| 2.85 (1.51-5.40)* \| \| 660 ≤ time < 3300 days \|  \| 1 \| 1.11 (0.69-1.77) \| 0.92 (0.56-1.52) \| 1.74 (1.08-2.81)† \| 1.32 (0.77-2.27) \| 2.21 (1.40-3.47)* \| \| time ≥ 3300 days \|  \| 1 \| 0.17 (0.06-0.49)* \| 0.28 (0.06-1.34) \| 0.36 (0.05-2.82) \| 0.34 (0.03-3.93) \| 0.58 (0.16-2.10) \| \| Diabetic foot \|  \| 1 \| 0.99 (0.36-2.76) \| 0.68 (0.25-1.88) \| 1.02 (0.37-2.83) \| 1.44 (0.54-3.83) \| 3.08 (1.32-7.19)* \| \| Data are presented as hazard ratio (95% confidential interval). A composite analysis of CKD development and progression was performed in CKD naïve group and pre-existing CKD group (n = 2769). Analysis for the development of ESRD was performed in total subjects (n = 3099). **p*<0.01, †*p*<0.05, adjusted for age, sex, body mass index, systolic blood pressure, high-density lipoprotein cholesterol, antihypertensive, and glucose-lowering agents  Abbreviations: CKD, chronic kidney disease; ESRD, end-stage renal disease; HR, hazards ratio; CI, confidence interval \| \| \| \| \| \| \| \| \| \| \| \| \| \| \| \| | | | | | | | |

**Electronic Supplementary Fig 1**. **Changes in baseline glycated hemoglobin (HbA_1c_) by the median at every two years.**

**
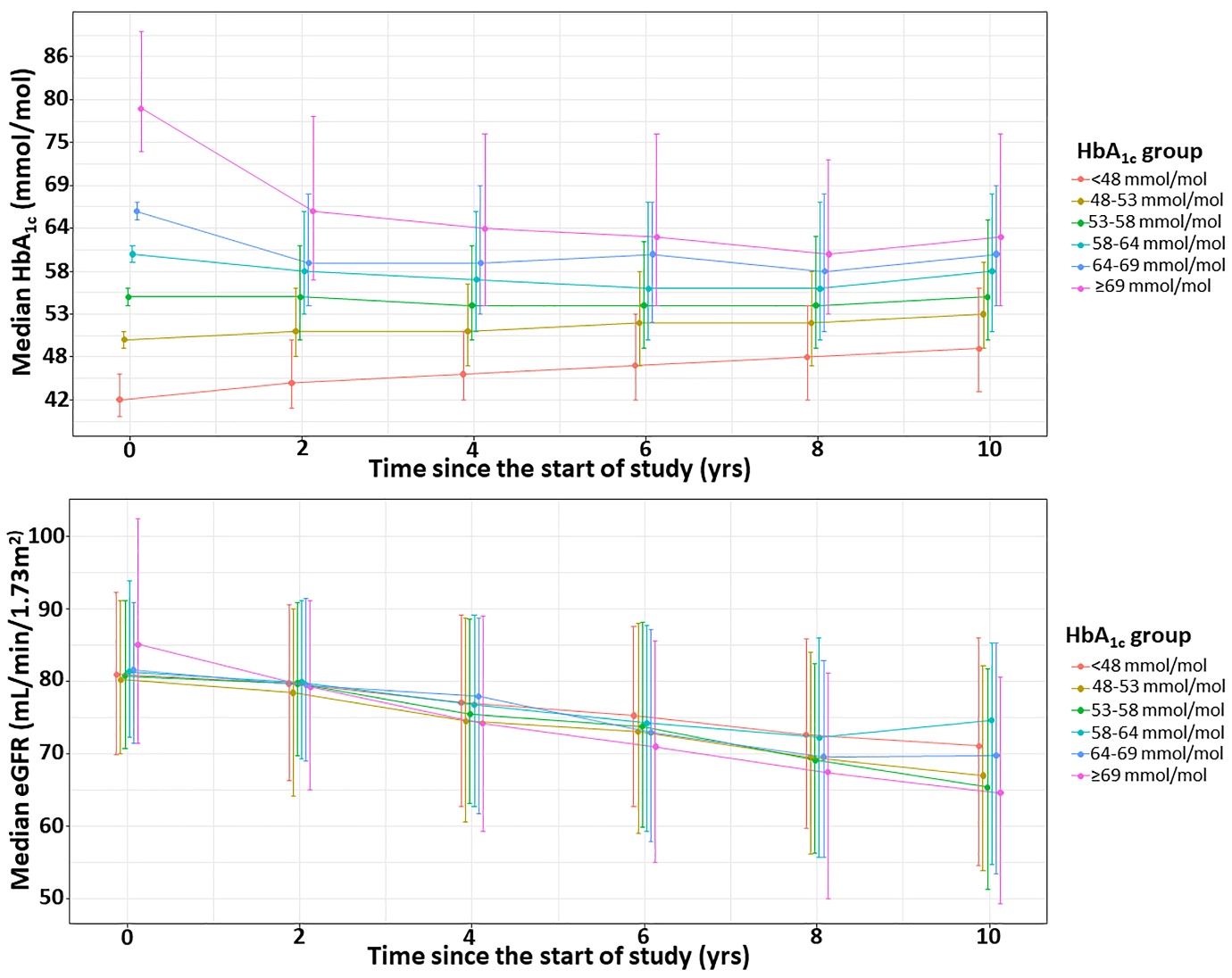
**

| Baseline HbA_1c_ | 0 | 2 yr | 4 yr | 6 yr | 8 yr | 10 yr |
| --- | --- | --- | --- | --- | --- | --- |
| <48 mmol/mol (6.5%) | 42.06 [39.88;45.34] | 44.25 [40.97;49.72]* | 45.34 [42.06;50.81]* | 46.44 [42.06;52.99]* | 47.53 [42.06;54.09]* | 48.62 [43.16;56.27]* |
| 48-53 mmol/mol  (6.5-7%) | 49.72 [48.62;50.81] | 50.81 [47.53;56.27]* | 50.81 [46.44;56.82]* | 51.90 [46.44;58.46]* | 51.90 [46.44;58.46]* | 52.99 [48.62;59.55]* |
| 53-58 mmol/mol  (7–7.5%) | 55.18 [54.09;56.27] | 55.18 [49.72;61.74] | 54.09 [49.72;61.74] | 54.09 [48.62;62.29] | 54.09 [48.62;62.83] | 55.18 [49.72;65.02]* |
| 58-64 mmol/mol  (7.5–8%) | 60.65 [59.55;61.74] | 58.46 [52.99;66.11] | 57.37 [50.81;66.11]* | 56.27 [49.72;67.21]* | 56.27 [49.72;67.21]* | 58.46 [50.81;68.30] |
| 64-69 mmol/mol  (8–8.5%) | 66.11 [65.02;67.21] | 59.55 [54.09;68.30]* | 59.55 [52.99;69.39]* | 60.65 [51.90;67.21]* | 58.46 [50.81;68.30]* | 60.65 [54.09;69.39]* |
| >69 mmol/mol  ≥8.5% | 79.23 [73.76;89.07] | 66.11 [57.37;78.14]* | 63.93 [54.09;75.95]* | 62.83 [54.09;75.95]* | 60.65 [52.99;72.67]* | 62.83 [54.09;75.95]* |

*indicates that there is a significant difference when comparing the baseline HbA_1c_ and the HbA_1c_ value of the corresponding period in each HbA_1c_ group.

**Electronic Supplementary Fig 2. Kaplan-Meier curves for the development or progression of chronic kidney disease using dynamic glycated hemoglobin level**


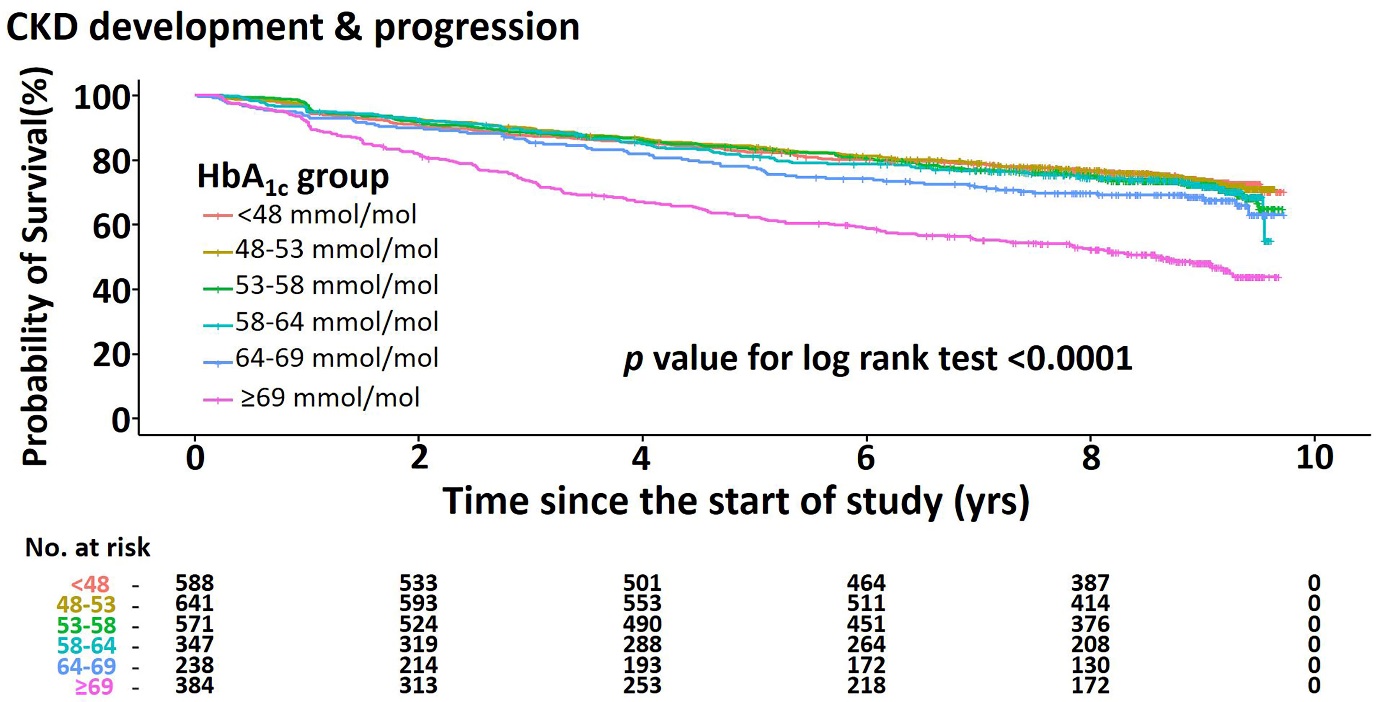


Abbreviations: HbA_1c_, glycated hemoglobin; CKD, chronic kidney disease; No, number

Kaplan-Meier curves stratified by dynamic HbA_1c_ into six groups. Renal outcome was defined as a composite event of the first CKD development in the CKD naïve group (n=2357) and CKD progression in the preexisting CKD group (n=412).

**Electronic Supplementary Fig 3. Kaplan-Meier curves for the development of end-stage renal disease using dynamic glycated hemoglobin level**


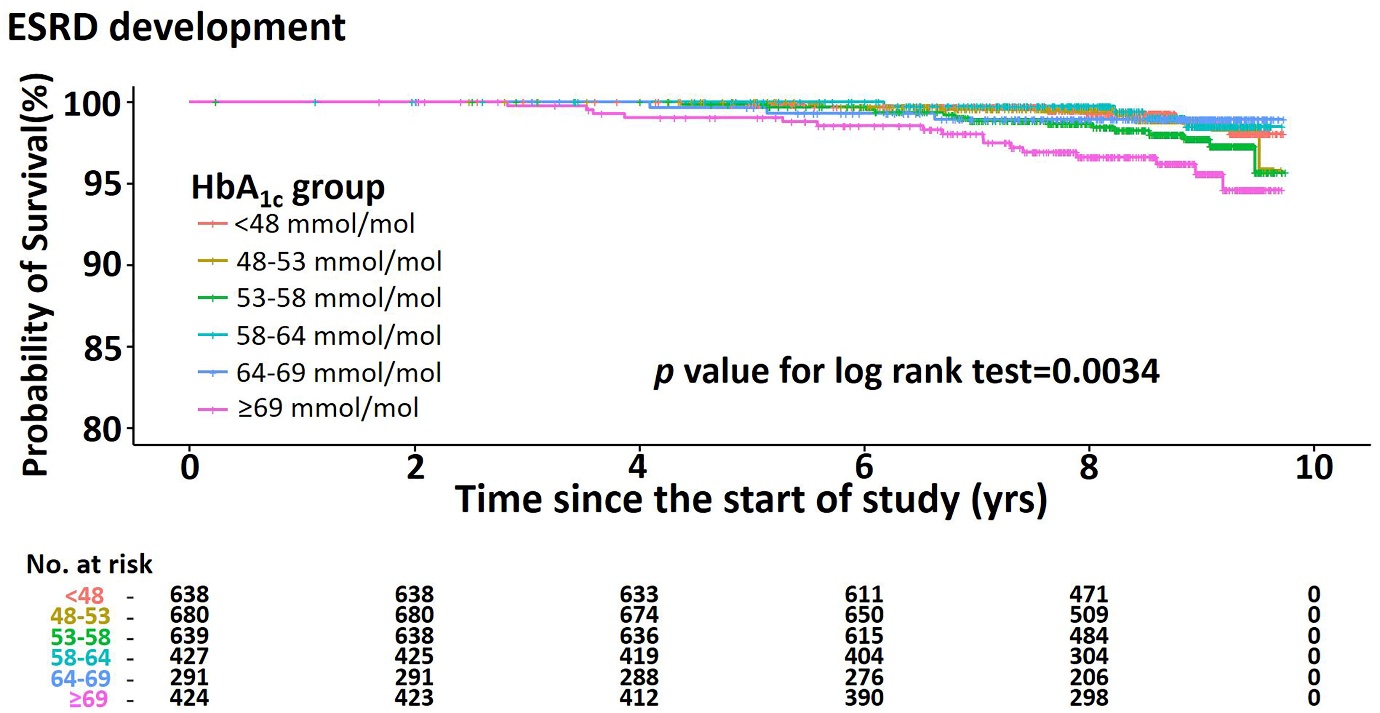


Abbreviations: HbA_1c_, glycated hemoglobin; ESRD, end-stage renal disease; No, number

Kaplan-Meier curves stratified by dynamic HbA_1c_ into six groups. Renal outcome was defined as the first ESRD development in all subjects including CKD naïve (n=2357), normal GFR with albuminuria (n=330), and preexisting CKD (n=412).
